# Delayed diagnosis of tuberculosis in patients with diabetes mellitus co-morbidity and its associated factors in Zhejiang Province, China

**DOI:** 10.1186/s12879-021-05929-8

**Published:** 2021-03-18

**Authors:** Wenhui Xiao, Dajiang Huang, Saiqiong Li, Shangcheng Zhou, Xiaolin Wei, Bin Chen, Guanyang Zou

**Affiliations:** 1 School of Public Health and Management, Guangzhou University of Chinese Medicine, Guangzhou, China; 2 Center for Disease Prevention and Control, Cangnan County, Wenzhou, Zhejiang Province China; 3 Center for Disease Prevention and Control, Yongjia County, Wenzhou, Zhejiang Province China; 4 Division of Clinical Epidemiology & Institute of Health Policy, Management and Evaluation, Dalla Lana School of Public Health, University of Toronto, Toronto, Canada; 5 Zhejiang Provincial Center for Disease Prevention and Control, Hangzhou, China

**Keywords:** Tuberculosis, Diabetes mellitus, Co-morbidity, Delay

## Abstract

**Background:**

Tuberculosis (TB) remains a significant global public health problem. China has the second highest TB burden in the world. With a growing TB population with diabetes mellitus (DM), the TB control system faces mounting challenges. To date, evidence remains inconclusive regarding the association between TB-DM co-morbidity and delayed diagnosis of TB patients. This study aims to assess the diagnostic delay of TB patients with known DM and identify the factors associated with this delay.

**Methods:**

Data was collected from China’s Tuberculosis information management system in two counties of Zhejiang province, China. Patient delay, health system delay and total diagnostic delay are defined as follows: 1) the interval between the onset of TB symptoms and first visit to any health facility; 2) from the first visit to the health facility to the confirmed TB diagnosis in the designated hospital; 3) the sum of patient and health system’s respective delays. Comparison of these delays was made between TB patients with and without DM using Mann-Whitney U test and Chi-square test. Univariate and multivariate regression analysis was used to identify factors influencing delays among TB patients with DM.

**Results:**

Of 969 TB patients, 67 (7%) TB patients had DM co-morbidity. Compared with TB patients without DM, TB patients with DM experienced significantly shorter health system delays (*p* < 0.05), and there was a significantly lower proportion of patients whose health system delayed> 14 days (7.0% vs. 18%, *p* < 0.05). However, no significant difference was observed between both patient categories regarding patient delay and total diagnostic delay. The multivariate regression analysis suggested that TB patients with DM who were aged < 60 years (AOR = 3.424, 95%CI: 1.008–11.627, *p* < 0.05) and non-severe cases (AOR = 9.725, 95%CI: 2.582–36.626, *p* < 0.05) were more likely to have a total diagnostic delay of> 14 days.

**Conclusions:**

Our study suggests that DM does not contribute to further diagnostic delay as expected. Instead, we observed significantly improved health system delay among TB patients with DM. The findings indicate the importance of early screening and diagnosis for TB among diabetic patients and of strengthening the integrated control and management of TB and diabetic programs.

## Background

Tuberculosis (TB) remains a significant global public health problem. In 2018, 10 million people were newly diagnosed with TB with 1.24 million deaths [1]. Early diagnosis and timely treatment of TB plays an important role in TB control, especially in controlling the spread of TB within the community [2] and avoiding poor disease prognosis [3]. However, health-care seeking delays in TB patients are common. Studies have reported the socioeconomic factors associated with delayed health-seeking among TB patients, such as older age, being female, low education level, and income [4–6] as well as clinic characteristics such as smear positive results, pulmonary cavity, cough, and night sweats [3, 7, 8].

Diabetes mellitus (DM) is a severe chronic disease characterized by hyperglycemia, which leads to disabling and life-threatening health complications [9]. Global figures for 2019 show that an estimated 463 million adults in the 20–79 age range live with DM, while the total number is predicted to rise to 578 million by 2030 [9]. Furthermore, the deaths attributed to DM and related complications in 2019 is estimated to surpass 4 million [9]. DM is an important risk factor for TB; people with DM were reported to have a threefold higher probability of getting TB compared to people without DM [10]. Worldwide research studies indicate that 1.9–50% of TB patients have DM [11, 12]. It is generally believed that TB is a disease of poverty, as 97% of TB cases were reported from 119 low-and middle-income countries in 2018 [1]. With DM co-morbidity, TB patients face even greater financial burden and the co-morbidity could seriously impoverish both patients and fragile health systems. TB patients with DM have to deal with more complicated problems than general TB patients such as contradictory dietary recommendations and low adherence to medication. The screening, treatment and care of patients with both TB and DM also require coordinated planning and service delivery across communicable and non-communicable disease programs [13]. Thus, the International Union against Tuberculosis and Lung Disease (IUATLD) issued guidelines for management of patients with TB-DM co-morbidity following the launch of a collaborative framework to advocate and implement joint care and control of TB and DM with the World Health Organization (WHO) in 2011 [14, 15].

China has the second highest TB burden in the world [1]. In 2018, an estimated 860,000 people fell ill with TB, accounting for 9% of the world’s TB case burden [1]. TB caused nearly 40,000 deaths in the same year. China also has one of the largest burdens in DM in the world, with 9.2% age-adjusted comparative prevalence and a population living with diabetes of 100 million in 2019 [9]. In a pilot project conducted in TB clinics /hospitals in China, the overall prevalence of DM in patients with TB was 12.4% [16]. Another community-based cohort study showed that TB patients had a higher odds ratio (OR: 3.17) of having DM than non-TB controls [17]. In most of China, and since the year 2000, TB patients receive standardized diagnosis and treatment in the TB clinic that is integrated in the county’s designated general hospital [18]. Other health facilities, including township hospitals and village clinics refer TB patients or presumptive TB patients to the designated TB hospitals for standardized diagnosis and treatment [18].

To date, most studies on TB and DM are epidemiological studies [11, 16, 17]. Very few studies have focused on the management of patients with TB-DM co-morbidity [13]. In addition, few studies examine delays in health-care seeking among TB-DM co- morbidity patients or explore factors that might explain these delays within this patient group. In an observational study conducted at community level, Wang et al. found that hyperglycemia was significantly associated with a higher risk of total diagnostic delay in TB patients over 30 years. This study also found that older age and lack of TB awareness are associated with a significantly higher risk of such delay in TB patients [3]. Chen et al. reported that DM was associated with a longer patient delay of TB patients in two TB dispensaries of Beijing, and smear positivity was positively associated with patient delay among TB patients with DM [7]. While both studies have reported that DM is associated with more serious diagnostic delay in TB patients [3, 7], more evidence is needed to verify this relationship. This study examines delayed diagnosis of TB patients with DM as compared to those without DM and identifies factors causing these delays to provide the evidence base for improved case detection and management of TB patients with DM co-morbidity.

## Methods

### Study design

This is a retrospective study of delayed diagnosis of TB patients with DM as compared to those without DM. Data were collected from patient records exported from China’s Tuberculosis information management system (TBIMS, TB special reporting system version 2.0).

### Study setting

This study was conducted in Cangnan and Yongjia, two counties of Wenzhou City, Zhejiang province. Table 1 shows the socioeconomic, demographic and health service information for the two counties under study and Wenzhou city. The permanent population of both studied counties are 1.35 million and 0.98 million respectively (8.25 million in Wenzhou City). Both counties have an average per capita GDP of US $5677 and $5672 in 2017 respectively, lower than that of Wenzhou city ($9761), and much lower than that of Zhejiang province($13,638). Similarly, the per capita disposable income for both counties is also lower than that of Wenzhou City and Zhejiang Province [19, 20]. The number of beds, practicing (assistant) physicians, and registered nurses per 1000 people in the two counties are also lower than that of Wenzhou city [19] (Table 1). In both counties, there is a designated general hospital where all TB cases are either referred to or self-presented for standardized diagnosis and treatment.

**Table 1:** Socio-economic and demographic, health service characteristics of Cangnan and Yongjia counties and Wenzhou City (2017)

	Wenzhou	Cangnan county	Yongjia county
Permanent population (million, people)^a^	8.25	1.35	0.98
GDP per capita (US $)^a^	9761	5677	5672
Per capita disposable income of urban residents (US $)	7684	6438	6187
Per capita disposable income of rural residents (US $)	3727	3005	3003
Number of medical and health institutions	5579	750	552
Number of beds in medical and health institutions (per 1000 people)	4.84	3.54	3.26
Number of practicing (assistant) physicians (per 1000 people)	3.23	1.92	2.41
Number of registered nurses (per 1000 people)	3.15	2.03	1.89

Note: 1 US $ = 6.75 RMB

^a^Wenzhou Prefectural Bureau of Statistics, http://wztjj.wenzhou.gov.cn/art/2018/11/14/art_1468704_24611681.html

### Data collection

This study was coordinated by Center for Disease Prevention and Control (CDCs) of Zhejiang Province, Cangnan CDC and Yongjia CDC. The CDC staff in these two counties exported patient data in 2017 from TBIMS to the Microsoft Excel (Microsoft, Redmond, WA, USA). Data covers general characteristics of TB patients such as age, sex, household registration status, level of hospital for initial TB diagnosis; clinical information such as TB severity (e.g. with large cavities or lesions in more than two lung lobes), cavity, treatment duration, smear sputum results, treatment classification, DM status; and health service-related data such as time of onset of TB symptoms, time of first health-care visit, time of confirmed TB diagnosis. Most of these data (including DM status) were collected and recorded during TB consultation by the TB health workers at the time of TB registration. DM is routinely screened through self-report of TB patients during TB consultation, and laboratory examination of blood sugar and glycated hemoglobin (HbA1C) is not compulsory.

### Definitions

In this paper, we study patient delay, health system delay and total diagnostic delay. Patient delay is defined as the interval between the onset of TB symptoms and first visit to a health facility. Health system delay is defined as the interval between the first visit to a health facility and confirmed TB diagnosis in the TB designated hospital. Total diagnostic delay (hereinafter referred to as total delay) is defined as the interval between the onset of TB symptoms and TB confirmation diagnosis, which is the sum of the patient delay and health system delay. In the meantime, we use 14 days as a cut-off point for analysis of patient delay, health system delay and total delay based on previous studies [21, 22]. Diagnosis is mainly based on sputum smear examination, supplemented by sputum culture and X-Ray. Patients with TB, patients with presumptive TB, and those with presumptive TB symptoms would have sputum checked three times, that is, to examine a sample of “instant sputum” in the outpatient clinic on the same day, and “night sputum” and “morning sputum” for examination the next day [23].

### Data analysis

Data was analyzed using SPSS 21.0 (SPSS, Inc., Chicago, USA). Descriptive statistics were adopted to report general and delay characteristics of TB patients with and without DM (including patient delay, health system delay and total delay). Median and Interquartile Range (IQR) was used for continuous variables, while counts and proportions were used for categorical variables. The univariate analysis including Mann-Whitney U Test and Chi-square test was employed to compare general characteristics and delay between TB patients with and without DM, and identify factors that are associated with delay of TB patients with DM. The multivariate regression analysis including linear regression and binary logistic regression was used to confirm factors associated with delay of TB patients with DM. The dependent variables were number of days of patient delay, health system delay and total delay in the linear regression, and patient delay, health system delay and total delay > 14 days (Yes = 1, No = 0) in the binary logistic regression, respectively. The independent variables were general and clinical characteristics of patients. The independent variables of delay with a *p* value < 0.2 in the Mann-Whitney U Test and Chi-square test were included in the subsequent multivariate regression analysis, using backward method, to adjust for potential confounding and identify those which were statistically associated with delay. The results were presented as adjusted ORs with 95% CI. The significant level was set at 5%.

## Results

### General and clinical characteristics of TB patients with DM as compared to those without DM

Of all 969 TB patients, 67 (7%) had TB-DM co-morbidity. The median (IQR) age was 47 (30–62), 27% were above 60 years, 70% were male, 66% were local residents, 36% were smear positive patients, 89% were newly registered patients, 23% were severe cases, 33% have cavity. The median (IQR) treatment duration was 330 (187–366) days, 81% received initial TB diagnosis at county-level hospital, 31% were cured and 61% completed treatment. The DM prevalence among male and female patients was 7.8% (53/681) and 4.9% (14/288), and no significant difference was found between these two groups.

Compared with TB patients without DM, TB patients with DM had significantly higher median age (58 vs. 46, *p* < 0.05), higher proportion of patients who were above 60 years (43% vs. 26%, *p* < 0.05), local residents (88% vs. 65%, *p* < 0.05), smear positive (51% vs. 35%, *p* < 0.05), new patients (100% vs. 88%, *p* < 0.05), severe cases(36% vs. 22%, *p* < 0.05), with cavity (54% vs. 32%, *p* < 0.05), and who had initially been diagnosed at county-level general hospitals (94% vs. 80%, *p* < 0.05) (Table 2).

**Table 2:** Socio-demographic and clinical characteristics of TB patients with and without DM

	All TB patients	TB patients with DM	TB patients without DM	Statistics /***P*** value
	(n=969)	(n=67)	(n=902)	
**Age (Median, IQR)**	47 (30–62)	58 (50–66)	46 (29–61)	Z = -5.403/ *P* < 0.001
**Age > 60y (n, %)**				χ^2^ = 8.921/*P* = 0.003
Yes	266 (27)	29 (43)	237 (26)	
No	703 (73)	38 (57)	665 (74)	
**Sex (n, %)**				χ^2^ = 2.684/ *P* = 0.101
Male	681 (70)	53 (79)	628 (70)	
Female	288 (30)	14 (21)	274 (30)	
**Household registration status (n, %)**				χ^2^ = 15.06/ *P* < 0.001
Local	644 (66)	59 (88)	585 (65)	
Migrant	325 (34)	8 (12)	317 (35)	
**Smear sputum results (n, %)**				χ^2^ = 7.091/ *P* = 0.008
Negative	623 (64)	33 (49)	590 (65)	
Positive	346 (36)	34 (51)	312 (35)	
**Treatment classification (n, %)**				χ^2^ = 9.123/*P* = 0.003
New patients	860 (89)	67 (100)	793 (88)	
Retreated patients	109 (11)	0 (0)	109 (12)	
**Severe cases (n, %)**				χ^2^ = 6.536/ *P* = 0.011
Yes	224 (23)	24 (36)	200 (22)	
No	745 (77)	43 (64)	702 (78)	
**Cavity (n, %)**				χ^2^ = 12.963/*P* < 0.001
Yes	324 (33)	36 (54)	288 (32)	
No	638 (66)	31 (46)	607 (68)	
**Treatment duration (Median, IQR)**	330 (187–366)	325 (190–370)	330 (187–366)	Z = -0.201/ *P* = 0.841
**Level of hospital for initial TB diagnosis (n, %)**				χ^2^ = 7.837/*P* = 0.005
County	786 (81)	63 (94)	723 (80)	
Prefectural	183 (19)	4 (6)	179 (20)	
**Reasons for stopping treatment (n, %)**				
Cured	303 (31)	31 (46)	272 (30)	
Complete treatment	587 (61)	31 (46)	556 (62)	
Lost	23 (2.4)	0 (0)	23 (2.5)	
Failed	20 (2.1)	1 (1.5)	19 (2.0)	
Died of TB	1 (0.1)	1 (1.5)	0 (0)	
Died of non-TB	6 (0.6)	2 (3.0)	4 (0.4)	
Transfer to MDR	7 (0.7)	1 (1.5)	6 (0.7)	
Adverse reactions	4 (0.4)	0 (0)	4 (0.4)	
Diagnostic change	14 (1.3)	0 (0)	13 (1.4)	
Other	5 (0.5)	0 (0)	5 (0.6)	

### Patient delay, health system delay and total delay of TB patients with DM as compared to those without DM

Of all 969 patients, the median (IQR) patient delay was 15 (5–35) days, while 52% had patient delay> 14 days. The median (IQR) health system delay was 0 (0–4) days, while 17% had system delay> 14 days. The median (IQR) total delay was 20 (7–48) days, while 61% had total delay> 14 days.

Compared with TB patients without DM, TB patients with DM experienced significantly shorter health system delays (0 vs. 0, *p* < 0.05), had significantly lower proportion of patients whose health system delay> 14 days (7.0% vs. 18%, *p* < 0.05). No significant difference was found between these two patient categories regarding patient delay (17 vs. 15, *p* > 0.05), total delay (19 vs. 21, *p* > 0.05), proportion of patients with patient delay (54% vs. 52%, *p* > 0.05) and total delay> 14 days (55% vs. 61%, *p* > 0.05) (Table 3).

**Table 3:** Patient, health system and total delay of TB patients with and without DM

	All TB patients	TB patients with DM	TB patients without DM	Statistics /***P*** value
	(n=969)	(n=67)	(n=902)	
**Patient delay (Median, IQR)**	15 (5–35)	17 (6–28)	15 (5–36)	Z = -0.497/*P* = 0.619^a^
**Patient delay > 14 d(n, %)**				χ^2^ = 0.085/*P* = 0.770
Yes	504 (52)	36 (54)	468 (52)	
No	465 (48)	31 (46)	434 (48)	
Health system delays (Median, IQR)^*^ **Health system delay > 14 d(n, %)**	0 (0–4)	0 (0–0)	0 (0–4)	Z = -4.197/*P* = 0.000^b^ χ^2^ = 4.897/ *P* = 0.027
Yes	168 (17)	5 (7.0)	163 (18)	
No	801 (83)	62 (93)	739 (82)	
**Total delay (Median, IQR)** **Total delay > 14 d(n, %)**	20 (7–48)	19 (7–32)	21 (7–50)	Z = -1.641/P = 0.101^c^ χ^2^ = 0.969/*P* = 0.325
Yes	590 (61)	37 (55)	553 (61)	
No	379 (39)	30 (45)	349 (39)	

^a^The mean rank of patient delay among TB patients with and without DM are 468.60, 486.22 respectively

^b^The mean rank of health system delays among TB patients with and without DM are 361.81, 494.15 respectively; ^c^ The mean rank of total delay among TB patients with and without DM are 430.87, 489.02 respectively

^*^ 575 patients (59%) out of all 969 patients had a health system delays of 0 day. TB patients with DM co-morbidity had higher proportion of health system delays of 0 day, as compared to those without DM co-morbidity (82% vs. 58%, **χ**^**2**^ = 15.568, *p* < 0.000)

In addition, TB patients with DM co-morbidity had higher proportion of health system delays of 0 day, as compared to those without DM co-morbidity (82% vs. 58%, *p* < 0.05, See Table 3).

### Factors influencing patient delay, health system delay and total delay of TB patients with DM

In TB patients with DM, univariate analysis showed that the variable of severe case or not was significantly associated with the number of days of patient delay (*p* < 0.05). The variables of household registration status and level of hospital for initial TB diagnosis (county-level or prefectural-level) were significantly associated with the number of days of health system delays (*p* < 0.05). The variables of age (> 60 years or not), household registration status, severe case or not and level of hospital for initial TB diagnosis (county-level or prefectural-level) were significantly associated with the number of days of total delays (*p* < 0.05). Further linear regression showed that, TB-DM patients who were initially diagnosed at prefectural-level hospital (AOR = 25.179, 95%CI: 14.698–35.659) tended to have longer health system delays (Table 4).

**Table 4:** Factors influencing the number of days of patient delays, health system delays and total delays of TB patients with DM

Variables	Patient delay (Median, IQR)			Health system delay (Median, IQR)			Total delay (Median, IQR)		
	Days	Adjusted β	95% CI	Days	Adjusted β	95% CI	Days	Adjusted β	95% CI
**Age > 60 y**									
Yes	12 (3–26)			0 (0–0)			12 (3–26)		
No	20 (7–29)	14.681	−5.111 to 34.474	0 (0–0)	−1.903	−7.154 to 3.358	22 (10–34)*	17.962	−2.580 to 38.504
**Sex**									
Male	16 (6–27)			0 (0–0)			17 (6–33)		
Female	22 (6–31)			0 (0–3)			22 (10–32)		
**Household registration status**									
Local	16 (6–25)			0 (0–0)			16 (6–25)		
Migrant	21 (8–57)			11 (0–30) ***	−0.316	−11.122 to 10.490	46 (26–65)*	17.556	−13.714 to 48.827
**Smear sputum results**									
Negativity	18 (4–30)			0 (0–1)			21 (6–33)		
Positivity									
**Treatment classification**									
New patients	17 (6–28)			0 (0–0)			19 (7–32)		
Retreated patients	/			/			/		
**Severe case**									
Yes	8 (5–22)			0 (0–0)			8 (5–23)		
No	20 (7–32)*	16.948	−3.472 to 37.369	0 (0–0)	−0.176	−7.604 to 7.253	21 (11–36)*	19.503	−1.689 to 40.694
**Cavity**									
Yes	14 (6–25)			0 (0–0)			14 (6–25)		
No	20 (5–31)			0 (0–1)	2.499	−2.536 to 7.535	21 (7–33)		
**Level of hospital for initial TB diagnosis**									
County	17 (6–29)			0 (0–0)			17 (6–30)		
Municipal	19 (8–21)			27 (19–35) ***	25.179	14.698 to 35.659***	46 (27–55)*	−22.844	−82.551 to 36.864

***P < 0.001, **p < 0.01, *p < 0.05

Similarly, univariate analysis showed that the variable of severe case or not was significantly associated with patient delay> 14 days (*p* < 0.05). The variables of household registration status, and level of hospital for initial TB diagnosis (county-level or prefectural-level) were significantly associated with health system delay> 14 days (*p* < 0.05). The variable of severe cases or not were significantly associated with the total delay> 14 days (*p* < 0.05). Further multivariate regression analysis showed that, TB patients with DM who were non-severe cases (AOR = 5.031, 95%CI: 1.696–14.918) were more likely to have patient delay> 14 days. TB patients with DM who were < 60 years (AOR = 3.424, 95%CI: 1.008–11.627), non-severe cases (AOR = 9.725, 95%CI: 2.582–36.626) were more likely to have total delay> 14 days (Table 5).

**Table 5:** Factors influencing the probability of patient delay, health system delay and total delay (> 14 days) of TB patients with DM

Variables	Patient delay (n, %)					Health system delay (n, %)				Total delay (n, %)		
	> 14 days	<=14 days	Adjusted OR	95% CI	> 14 days	<=14 days	Adjusted OR	95% CI	> 14 days	<=14 days	Adjusted OR	95% CI
**Age > 60 y**												
Yes	13 (36)	16 (52)			1 (20)	28 (45)			13 (35)	16 (53)		
No	23 (64)	15 (48)			4 (80)	34 (55)			24 (65)	14 (47)	3.424	1.008 to 11.627*
**Sex**												
Male	27 (75)	26 (84)			4 (80)	49 (79)			28 (76)	25 (83)		
Female	9 (25)	5 (16)			1 (20)	13 (21)			9 (24)	5 (17)		
**Household registration status**												
Local	30 (83)	29 (94)			1 (20)	58 (94)			30 (81)	29 (97)		
Migrant	6 (17)	2 (6)			4 (80)	4 (6) ***	**/**	**/**	7 (19)	1 (3)	10.312	0.886 to 120.03
**Smear sputum results**												
Negativity	19(53)	14 (45)			4 (80)	29 (47)			20 (54)	13 (43)		
Positivity	17(47)	17 (55)			1 (20)	33 (53)	/	/	17 (46)	17 (57)		
**Treatment classification**												
New patients	36 (100)	31 (100)			5 (100)	62 (100)			37 (100)	30 (100)		
Retreated patients	/	/			/	/			/	/		
**Severe case**												
Yes	7 (19)	17 (55)			1 (20)	23(37)			7 (19)	17 (57)		
No	29 (81)	14 (45) **	5.031	1.696 to 14.918 **	4 (80)	39 (63)			30 (81)	13 (43) ***	9.725	2.582 to 36.626**
**Cavity**												
Yes	16 (44)	20 (65)			1 (20)	35 (56)			16 (43)	20 (67)		
No	20 (56)	11 (35)	0.606	0.135 to 2.175	4 (80)	27 (44)			21 (57)	10 (33)	0.776	0.158 to 3.819
**Level of hospital for initial TB diagnosis**												
County	33 (92)	30 (97)			1 (20)	62 (100)			33 (89)	30 (100)		
Municipal	3 (8)	1 (3)			4 (80)	0 (0) ***	/	/	4 (11)	0 (0)	/	/

***P < 0.001, **p < 0.01, *p < 0.05

## Discussion

### Summary of findings

In the present study, we found that of 969 TB patients, 7% patients had TB-DM co- morbidity, and TB patients with DM tended to be of older age, local residents, smear positive patients, new patients, severe cases and have cavity. The median total delay of TB patients with DM was 19 days, as compared to 21 days for TB patients without DM. Compared with TB patients without DM, TB patients with DM experienced shorter health system delays and lower probability of health system delay> 14 days. TB patients with DM who were aged< 60 years, non-severe cases were more likely to have total diagnostic delay > 14 days.

### Comparison with literature

In recent years China has undergone a rapid increase in DM burden in the context of high TB burden, thus, the TB control system faces a double challenge posed by TB- DM co-morbidity. Our study found that prevalence of DM in TB patients was 7%, which is similar to prevalence reported in Wang et al.’s community-based cohort study (6.3%) [17], but much lower than that in Li et al.’s study (12.4%) [16]. One possible explanation for the inconsistency in different DM prevalence studies is that our analysis is based on the data recorded from routine TB clinical consultations, where DM information is collected based on TB patients’ self-report and blood sugar screening is not routinely conducted among TB patients. In Wang et al.’s study, most TB patients were screened for DM in 2–3 weeks after the initiation of TB treatment, to avoid a potential over-diagnosis of hyperglycemia induced by TB temporarily [17]. It is noteworthy that DM prevalence among male TB patients was higher than that among female patients (7.8% vs. 4.9%), although without significant difference. It is worth exploring the underlying causes of this pattern of prevalence variation, taking into consideration, for example, the sex factor or access variables between male and female patients.

It was previously reported that DM patients are susceptible to lower respiratory tract infections [3]. The frequent symptoms of cough, fever, and chills would overlap typical TB symptoms, resulting in longer health-seeking delays in TB patients with DM. However, contrary to previous studies [3, 7], we found that TB patients with DM are associated with shorter patient delay and total delay as compared to TB patients without DM (although without statistical significance). One possible explanation for this is that TB patients with DM, especially when there was a higher proportion of severe cases among them as compared to those without DM in our study, may tend to seek health services earlier as they suffer from more serious clinical symptoms. In addition, we found significantly shorter health system delay among TB patients with DM as compared to TB patients without DM. This pattern is also true for the proportions of patients whose patient, health system and total delays were > 14 days. The significantly improved health system delay for TB patients with DM may be partly due to the improved knowledge and awareness of risk factors (including DM) for TB and improved referrals of patients with high risk to TB among health providers. In some places, TB screening is conducted among high-risk populations, including the elderly and diabetic patients. However, health providers should also recognize other common risk factors to TB such as HIV, smoking, alcoholism, malnutrition [1], and provide timely screening, diagnosis of TB or referral to the TB designated hospital. It is noteworthy that we identified a higher proportion of TB patients with DM with a health system delay of 0 days. As they are likely to seek diabetic care in the same general hospital where the designated TB clinic is located, it becomes easier for them to receive an internal referral to the TB clinic, where they are diagnosed and treated without further delays. Our study suggests the importance of health education and promotion to improve risk awareness for TB among diabetic patients, since we did not find significant difference between these two categories of patients with regards to patient delay and total diagnostic delay (which is mainly contributed by the patient delay). However, although we did not observe more serious delay in TB patients with DM, one should not neglect the challenges of managing patients with TB-DM co-morbidity [13].

Very few studies focused on factors affecting delay of patients with TB-DM co- morbidity. Chen et al. reported that smear positivity was positively associated with patient delay> 30 days for TB patients with DM [7]. Other studies have also reported that a higher risk of patient delay> 14 days was associated with smear positivity for TB patients [8, 24]. However, we did not observe that smear positivity had significant influence on patient delay among TB patients with DM in the present study.

Previous studies have reported that TB patients with mild TB symptoms, particularly those without hemoptysis, were more likely to have patient delay [3, 25]. Similarly, both of our univariate and multivariate regression analysis suggests that non-severe cases tended to have longer patient delay, total delay as well as a higher risk of patient delay and total delay > 14 days. It may be because severe cases may seek health services earlier as they suffered more serious clinical symptoms, while the non-severe cases tend to delay their care seeking due to milder symptoms. These findings suggest that intensive TB case finding among diabetic patients is important for early detection of TB cases, especially because some TB patients may be asymptomatic or have mild symptoms of TB, making it difficult to detect them by other means.

Similar to previous studies on migrant TB delay [26–29], our study showed that migrant TB patients with DM tended to have longer patient delays and a higher proportion of patients with patient delay> 14 days (although without statistical significance). This may be due to poor health awareness and health behavior among TB-affected migrants because of their poor socioeconomic characteristics, such as job instability, low income, poor living and working conditions [30–34]. In addition, our univariate analysis suggests that migrant TB patients with DM tended to have longer health system delays, total delay and a higher risk of health system delay> 14 days. This may be because migrants are mostly uninsured and floating and are less likely to follow the recommendation of timely referral [24]. Our study highlights the need to strengthen health education and referral and address financial barriers among migrant patients with DM, especially those with typical TB symptoms.

Similar to a previous study reporting delay in TB patients in China [35], our study showed that TB patients with DM who received initial TB diagnosis at a higher-level hospital (i.e. prefectural-level hospital) tended to have longer health system delays based on the multivariate regression analysis, and longer total delay and higher risk of health system delay> 14 days based on the univariate analysis. This suggests the challenge of timely referral of TB suspects or patients from the general especially tertiary hospitals to the TB program [36]. Co-morbidity with DM adds to this challenge as diabetic patients often seek care in tertiary hospitals. As a previous study in China showed, many patients made repeat visits to the prefectural-level hospital before being classified as having presumptive TB and referred to a TB-designated hospital for confirmed TB diagnosis [37]. It is, thus, important to improve monitoring and referral of persons with presumptive TB, especially among diabetic patients from higher-level health services to the TB designated hospital.

Our univariate and multivariate regression analysis suggests that TB patients with DM who were aged < 60 years endured longer total delay and have higher risk of total delay> 14 days, while other studies found older age is a risk factor for total delay [3]. One plausible reason is that patients < 60 years are of working age, and so it is difficult for them to take time away from work to visit health services [4].

### Limitations

Our study has several limitations. First, as a case study, generalizability of our findings is limited but it provides the basis to undertake a larger-scale cohort study, which would help us better understand the multiple factors influencing delays of patients with TB- DM co-morbidity. Second, due to constraints of the routine practice database, we could not include further basic social-economic indicators like education and income level in the analysis. These factors could also have influence on TB patients’ health-seeking behavior [4–6]. Third, under-detection of DM among TB patients is possible, since we did not conduct blood sugar screening among TB patients but mainly based on patients’ self-report of the DM conditions in the routine TB practices. Finally, the comparison may be biased as we have a low proportion of TB patients with DM, but this case study provides an initial understanding of the delay characteristics of TB patients with DM and associated factors.

## Conclusions

Our study suggests that DM does not contribute to further diagnostic delay as expected. Instead we observed significantly improved health system delay among TB patients with DM, although we did not find significantly reduced patient and total diagnostic delay among this patient group as compared to those without DM. Findings indicate the importance of early screening and diagnosis for TB among diabetic patients and of strengthening the integrated control and management of TB and diabetic programs.

## Data Availability

Information in our database is confidential, however, data used for the analysis is available upon reasonable request from corresponding author.

## Abbreviations

TB: Tuberculosis
DM: Diabetes Mellitus
IUATLD: The International Union against Tuberculosis and Lung Disease
WHO: World Health Organization
TBIMS: China's Tuberculosis information management system
IQR: Interquartile Range
ORs: Odds ratio
CIs: Confidence intervals
AOR: Adjusted odds ratio
CDCs: Center for Disease Prevention and Control
HbA1C: Glycated hemoglobin
